# A Stability-Indicating HPLC-DAD Method for Determination of Stiripentol: Development, Validation, Kinetics, Structure Elucidation and Application to Commercial Dosage Form

**DOI:** 10.1155/2014/638951

**Published:** 2014-10-14

**Authors:** Hany W. Darwish, Ali S. Abdelhameed, Mohamed I. Attia, Ahmed H. Bakheit, Nasr Y. Khalil, Abdulrahman A. Al-Majed

**Affiliations:** ^1^Department of Pharmaceutical Chemistry, College of Pharmacy, King Saud University, P.O. Box 2457, Riyadh 11451, Saudi Arabia; ^2^Analytical Chemistry Department, Faculty of Pharmacy, Cairo University, Kasr El-Aini Street, Cairo 11562, Egypt; ^3^Department of Medicinal and Pharmaceutical Chemistry, Pharmaceutical and Drug Industries Research Division, National Research Centre, Dokki, Giza 12622, Egypt

## Abstract

A rapid, simple, sensitive, and accurate isocratic reversed-phase stability-indicating high performance liquid chromatography method has been developed and validated for the determination of stiripentol and its degradation product in its bulk form and pharmaceutical dosage form. Chromatographic separation was achieved on a Symmetry C18 column and quantification was achieved using photodiode array detector (DAD). The method was validated in accordance with the ICH requirements showing specificity, linearity (*r* ^2^ = 0.9996, range of 1–25 *μ*g/mL), precision (relative standard deviation lower than 2%), accuracy (mean recovery 100.08 ± 1.73), limits of detection and quantitation (LOD = 0.024 and LOQ = 0.081 *μ*g/mL), and robustness. Stiripentol was subjected to various stress conditions and it has shown marked stability under alkaline hydrolytic stress conditions, thermal, oxidative, and photolytic conditions. Stiripentol degraded only under acidic conditions, forming a single degradation product which was well resolved from the pure drug with significantly different retention time values. This degradation product was characterized by ^1^H-NMR and ^13^C-NMR spectroscopy as well as ion trap mass spectrometry. The results demonstrated that the method would have a great value when applied in quality control and stability studies for stiripentol.

## 1. Introduction

**Figure 1:**
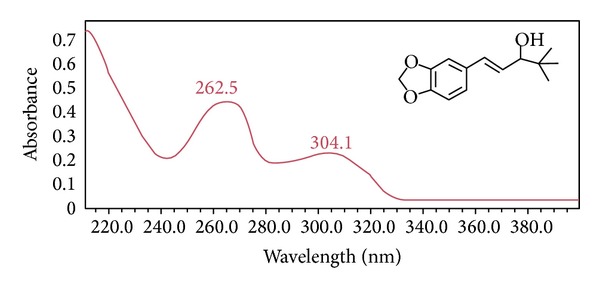
Chemical structure of stiripentol (STP) and its absorption spectrum against methanol. Concentration of STP was 10 *μ*g/mL in methanol.

Stiripentol (STP; (1*E*)-1-(1,3-benzodioxol-5-yl)-4,4-dimethylpent-1-en-3-ol; Figure 1) is a newly approved orphan antiepileptic drug (AED) [1, 2]. A characteristic feature of the drug is the presence of a chiral center at C-3. STP is marketed as a racemic mixture of two enantiomers:* R*(+)-STP and* S*(−)-STP [3] of which* R*(+)-STP has about 2.4-fold greater anticonvulsant potency and it is also associated with ~3-fold faster elimination rate than* S*(−)-STP [4]. It is structurally unrelated to any other currently available antiepileptic drugs [5]. The compound has been under investigations for nearly 4 decades [6, 7]; its clinical development was delayed due to the inhibitory effect of STP on hepatic cytochrome-P450 (CYP-450) [8]. Stiripentol, (Diacomit; Biocodex Inc.) has been approved by the European and Canadian marketing authorization for the treatment of Dravet syndrome or severe myoclonic epilepsy in infancy (SMEI) in conjunction with sodium valproate and clobazam when seizures are not adequately controlled with the association of these two medications [9, 10]. Its primary mode of action is* via* GABA inhibitory neurotransmission in the brain. STP inhibits GABA metabolism through the blockade of GABA-transaminase activity [11] and reduces synaptosomal uptake of GABA, leading to an increase in GABA brain content [12]. STP has, however, no affinity for GABA_A_ and GABA_B_ receptors [11]. STP, at relevant clinical concentrations (30–300 *μ*M), markedly increases the mean open duration of GABA_A_ receptor dependent chloride channels by a barbiturate-like mechanism [13].

Stability testing of pharmaceutical dosage form of STP or any other drug, (i.e., purity and stability of the active ingredient and/or final product) is imperative for the optimum and efficient delivery of its therapeutic activity to the patients. The purpose of stability testing is to investigate changes in the quality of a drug substance or a drug product with time under the influence of environmental factors, such as temperature, humidity, and light. Moreover, stability testing establishes the recommended storage conditions and shelf life for the drug product [14, 15]. This is actually due to the fact that presence of impurities and/or potential degradation products may alter the pharmacological and/or toxicological characteristics of the active drug [16, 17]. In particular, pharmaceuticals are significantly sensitive to environmental conditions, which are usually varied during the different stages (i.e., manufacturing, transportation and storage) of the finished products. Based on the aforementioned importance of stability testing, it is vital to investigate degradation pathways and the intrinsic stability of the drug, compatibility of the drug with the excipients in the dosage form, and the long-term effects of the environment. Recently, the need of establishing a stability-indicating assay method for the stability testing has become more clearly mandated in the official guidelines at the International Conference on Harmonization (ICH) [15] and United States Pharmacopeia (USP) [14]. A thorough literature survey revealed that only few high performance liquid chromatography (HPLC) methods were reported for the determination of STP to study its pharmacokinetics [7, 18], enantioseparation [19], and its estimation in plasma [20]. Consequently, a stability-indicating method for determination of STP in its bulk drug and pharmaceutical capsules is important.

There is no previous research pertaining to explore stability testing of STP. Accordingly, the present study describes for the first time a comprehensive stability-indicating study for STP. This study aims to develop and fully validate a rapid, simple, and accurate HPLC-DAD method to quantify STP in the presence of its degradation product in bulk and pharmaceutical dosage form. Additionally, structure elucidation techniques including the ^1^H-NMR, ^13^C-NMR, and mass spectrometry were used to identify STP degradation product.

## 2. Experimental

### 2.1. Chemicals and Reagents

Stiripentol reference standard (purity ~99.6%) was purchased from Sigma-Aldrich Co. (St. Louis, MO, USA). Diacomit capsules (Biocodex, Montrouge, France), labeled to contain 250 mg (as the anhydrous base) per capsule, were procured from the local market. HPLC-grade solvents and reagent-grade sodium hydroxide, hydrochloric acid, hydrogen peroxide, potassium dihydrogen phosphate, and orthophosphoric acid were purchased from Merck (Darmstadt, Germany). Ultrapure water of 18 *μ*Ω was obtained from a Millipore Milli-Q UF and purification system (Millipore, Bedford, MA, USA) was used throughout the study.

### 2.2. Chromatographic System

HPLC apparatus consists of a Waters breeze system consisting of 1525 binary pump with online degasser, 717+ autosampler, 5CH thermostated column compartment, and a 2996 photodiode array detector (DAD) (Waters Corporation, Milford, MA, USA). The chromatographic separations were performed on a Symmetry C18 column (3.5 *μ*m, 75 mm × 4.6 mm i.d) manufactured by Waters Corporation, Milford, MA, USA. The column temperature was kept constant at 25 ± 2°C. Separations were performed in isocratic mode using a mobile phase consisting of acetonitrile and 50 mM potassium dihydrogen phosphate buffer (60 : 40, v/v), and pH was adjusted to 4.1 ± 0.1 with 10% phosphoric acid solution. The mobile phase was filtered through a Millipore vacuum filtration system equipped with a 0.45 *μ*m filter (Millipore, Bedford, MA, USA), degassed by ultrasonic water bath prior to its use. The flow rate of the mobile phase was 1 mL/min, and the sample injection volume was 20 *μ*L. The DAD detector was set at scan range of 210–400 nm to allow investigation of any impurities and/or degradation products with shorter wavelength. Additionally, quantitation of STP and its acidic degradation product was performed at 262.5 nm. Prior to each run, the HPLC-DAD system was allowed to warm up for nearly 30 min and the baseline was monitored until it was stable before the samples were injected. Peak identity was confirmed by retention time comparison and comparison of the spectra obtained from the DAD detector.

### 2.3. Preparation of Standard and Sample Solutions

STP stock solution (1 mg/mL) was prepared by dissolving 25 mg of STP reference standard material into 25 mL methanol in a 25 mL volumetric flask and completing the volume properly. This stock solution was later diluted with methanol to produce a working standard solution of 100 *μ*g/mL. Working solution was stored at 4°C until required for analysis.

### 2.4. Preparation of Calibration Samples

An eight-point calibration curve (1, 2, 4, 6, 10, 15, 20, and 25 *μ*g/mL) was constructed by plotting the peak area of STP (*y*) versus STP nominal concentration (*x*). Analysis of calibration samples at each concentration was performed in triplicates. Slope, intercept, and *r* ^2^ values were calculated as regression parameters by linear regression. The linear regression equation was used to calculate the concentrations of STP in aqueous solutions and dosage form based on their peak area. Sample solutions were prepared by diluting the working solution with mobile phase to obtain final concentrations of STP.

### 2.5. Preparation of Capsule Samples

Twenty capsules (Diacomit 250 mg capsules; batch number 2611) were weighed and average weight was determined. Capsules were emptied and two portions (250 mg each) were separated where one portion was transferred to a 500 mL volumetric flask. A volume of 250 mL of methanol was added, the contents were mechanically shaken for 10 min and ultrasonicated for 5 min, and the volume was diluted to 500 mL with methanol. This solution (0.5 mg/mL) was diluted as required for analysis. The remaining 250 mg powder was used as it is for thermal and photodegradation studies.

### 2.6. Forced Degradation Procedures [15]

#### 2.6.1. Thermal Stress

Thermal degradation of the drug substance in its bulk form and capsules was carried out in both solid and solution state. Powder samples of standard STP (10 mg) and Diacomit capsules (10 mg of capsule content) were kept in a controlled-temperature oven at 80°C for 48 hours. These powder samples were separately dissolved in 10 mL methanol. Solutions were then diluted with mobile phase to obtain a concentration of 10 *μ*g/mL, and a volume of 20 *μ*L of each solution was injected into the HPLC system. Meanwhile, a volume of 1 mL of STP working solution (100 *μ*g/mL) and capsule extract solution (100 *μ*g/mL; prepared as previously mentioned in Section 2.5.) was transferred to small round bottom flasks and subjected to reflux at 80°C for 48 hours. The solutions were cooled to room temperature (25 ± 5°C) and diluted with mobile phase to yield final concentrations of 10 *μ*g/mL; and a volume of 20 *μ*L of each solution was then injected into the HPLC system.

#### 2.6.2. Acid/Base Forced Degradation

Aliquots of 1 mL of STP working solution (100 *μ*g/mL) and capsule extract solution (100 *μ*g/mL) were transferred to small round bottom flasks. In the preliminary forced degradation testing, it was observed that acid degradation takes place rapidly and the drug was almost fully degraded when 0.5 N hydrochloric acid was used. Therefore, in later experiment, acid degradation was performed with a maximum of 0.1 N hydrochloric acid. Ultimately, solutions were individually mixed with 4 mL of (0.005 N, 0.01 N, 0.05 N, and 0.1 N hydrochloric acid) and/or (0.05 N, 0.1 N, 0.5 N, and 1 N sodium hydroxide). The prepared solutions were heated for 1, 2, and 3 hours at 60°C in a water bath. The samples were cooled to room temperature (25 ± 5°C), neutralized with an amount of acid or base equivalent to that of the previously added. The resulting neutral solutions were then diluted with mobile phase to 10 *μ*g/mL and a volume of 20 *μ*L was injected into the HPLC system.

#### 2.6.3. Oxidative Degradation

Four aliquots of 1 mL STP working solution (100 *μ*g/mL) and capsule extract solution (100 *μ*g/mL) were transferred into small round bottom flasks. The contents were then mixed separately with 4 mL of (1, 6, 10, and 30%) hydrogen peroxide solutions, and the reaction mixtures were kept at ambient temperature for 2 hours with intermittent shaking. The resulting solutions were diluted with mobile phase to 10 *μ*g/mL and a volume of 20 *μ*L of each solution was injected into the HPLC system.

#### 2.6.4. Photodegradation

Powder samples of standard STP (10 mg) and Diacomit capsules (10 mg of capsule content) were UV irradiated (peak intensity of 254 nm) for 72 hours. Subsequently, these powders were dissolved in 10 mL methanol. Solutions were then diluted with mobile phase to obtain a concentration of 10 *μ*g/mL, and a volume of 20 *μ*L of each solution was injected into the HPLC system. Similarly, an aqueous solution of STP (1 mg/mL) and capsule extract solution (1 mg/mL) were exposed to the UV light for 72 hours and diluted with mobile phase to 10 *μ*g/mL and a volume of 20 *μ*L of each was then injected into the HPLC system.  Table 1 summarizes the forced degradation conditions for STP.

### 2.7. Structure Elucidation of Acidic Degradation Product of STP

#### 2.7.1. Sample Preparation

Acid degradation was carried out by refluxing STP (40 mg) in methanol (15 mL) and 8 N HCl (5 mL) for 3 h at 60°C using the same procedure discussed in Section 2.6.2. The resulting neutral solution was then concentrated under reduced pressure. The acid degradation product (DSTP) was extracted from the residue using diethyl ether which was subsequently evaporated under reduced pressure. Crude DSTP was then purified* via* column chromatography (silica gel 60; 0.063–0.200 mm) using chloroform as a solvent. The collected eluate was evaporated under vacuum to give pure DSTP, which was identified through  ^1^H-NMR, ^13^C-NMR, and mass spectrometry.

#### 2.7.2. NMR Analysis

Purified DSTP was dissolved in deuterated chloroform and the NMR measurements were performed at 25°C on a Bruker AC-500 NMR spectrometer operating at 500 MHz for ^1^H and 125.76 MHz for ^13^C. Tetramethylsilane (TMS) was used as an internal standard and chemical shift values were recorded in ppm on the *δ* scale. The ^1^H-NMR data were represented in terms of chemical shifts, multiplicity, and number of protons. The ^13^C-NMR data were represented as chemical shifts and type of carbon.

#### 2.7.3. Mass Spectrometric Analysis

For structure elucidation of DSTP, an Agilent 6410 triple quadrupole mass spectrometer (Agilent technologies, USA) equipped with an electrospray ionization interface (ESI) coupled to an Agilent 1200 HPLC (Agilent Technologies, USA) was used. Agilent 1200 series system consists of G1311A binary pump, G1322A degasser, and G1367B HIP-ALS autosampler, and G1316A thermostated column compartment was used. Purified DSTP was dissolved in acetonitrile (5 *μ*g/mL), and a connector was used instead of the column to allow direct injection of the sample. Mobile phase composed of two solvents: A is HPLC grade water and B is acetonitrile mixed in the ratio 50 : 50 v/v%. MS parameters were optimized to scan in ultrascan mode. For screening of mass signals of DSTP, MS2 scans were performed in the mass range of* m/z* 50–400. The ESI was operated in positive ion mode. The source temperature was set to 350°C nebulizer gas pressure of 55 psi and dry gas flow rate of 12 L/min.

### 2.8. Kinetic Investigation of STP Acidic Degradation

Four sets of STP working solution (100 *μ*g/mL) and capsule extract solution (100 *μ*g/mL), 1 mL of each, were subjected to acid degradation using 4 mL of 0.005 N, 0.01 N, 0.05 N, and 0.1 N hydrochloric acid (procedure described in Section 2.6.2). The flasks were heated at 60°C for specified time intervals (0, 15, 30, 60, 120, 180, and 240 min). The samples were cooled to room temperature (25 ± 5°C) and neutralized with an amount of base equivalent to that of the previously added acid. The resulting neutral solutions were then diluted with mobile phase to 10 *μ*g/mL and a volume of 20 *μ*L was injected into the HPLC system and the peak area of STP was compared with a freshly prepared standard solution. The remained STP concentration was calculated and plotted against time. Each experiment was repeated three times at each HCl concentration.

## 3. Results and Discussion

### 3.1. Method Development

**Figure 2:**
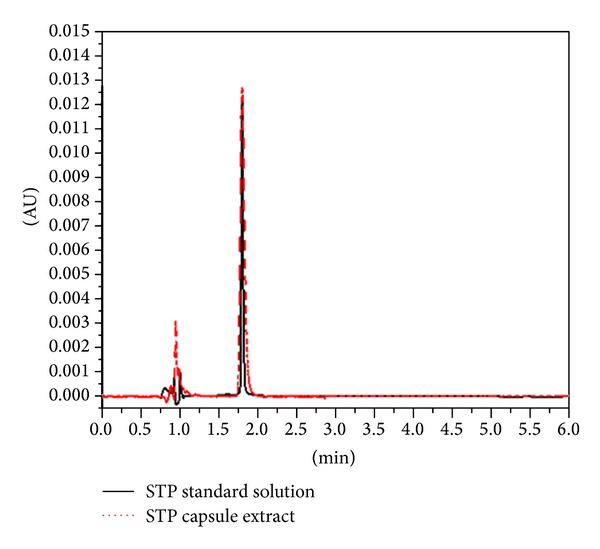
A representative chromatogram of 10 *μ*g/mL of untreated standard solution of STP (solid black line) and solution of capsule extract containing nominal STP concentration of 10 *μ*g/mL (dotted red tracing).

The initial method development was conducted on pure drug using working standards solution protected from light. Optimized chromatographic conditions were established after a number of preliminary experiments for selecting the most efficient mobile system, separation column, and detection wavelength range. Selection of the proper system was based on its ability to give good separation between the pure STP and its possible impurities and/or degradation products. Different mobile phase compositions and pH ranges were tested to achieve a more symmetric peak and shorter retention time for STP. Mobile phase was modified between 20 : 80% and 70 : 30% acetonitrile: 1 mM phosphate buffer and pH was changed in the range of 3–7. Ultimately, optimum chromatographic separation was achieved as described in Section 2.2. Under these chromatographic conditions, the run time of each sample was 6 min, and the retention time of STP was 1.80 ± 0.07 min (*n* = 3) (Figure 2). An advantage of the current method is its simple sample preparation as the procedure did not include significant error-prone experimental manipulations (e.g., chemical derivatization, etc.) which would negatively affect the results. This supported the avoidance of internal standard use, which is usually added to increase the method efficiency when there are multisteps in sample preparation.

### 3.2. Method Validation

The method was fully validated in accordance with the recommendations of ICH [15] and is rugged and adequately sensitive for routine STP analysis.

#### 3.2.1. Linearity and Sensitivity

Using the above-mentioned optimum chromatographic conditions, three independent calibration curves were constructed correlating the detector signals with the corresponding STP concentrations. Each curve was generated by 8 concentration points; each concentration was injected in triplicates. Regression analysis for the results was carried out using the least-square method. The standard deviation values of each concentration point (triplicates) did not exceed 2%. The method revealed a good linear calibration fit in the range of 1–25 *μ*g/mL. The *r* ^2^ value was 0.9996 indicative for the good linearity.

System suitability tests for the principle peak and its degradation product were evaluated using STP solution of 12 *μ*g/mL. The capacity factor *K* = 0.8884 indicates that the STP peak is well resolved with respect to the void volume. The asymmetry factor at 5% of peak height (*b*/*a*) was 1.3079 for STP peak which reflects good peak symmetry. Theoretical plate number (*N*) of 5008.09 for the column used in the study (3.5 *μ*m, 75 mm × 4.6 mm i.d) thus demonstrates acceptable column efficiency. Asymmetry at 10% of peak height was 0.944. All previous results assured the adequacy of the proposed HPLC method for routine analysis of STP.

#### 3.2.2. The Limit of Detection (LOD) and Limit of Quantitation (LOQ)

The lower limit of quantitation (LOQ) is the lowest concentration of the standard curve which can be measured with acceptable accuracy and precision for the analyte. The limit of detection (LOD) and the lower limit of quantitation (LOQ) were calculated based on the following equations:

(1) $$\begin{array}{c} \text{LOD}=3.3\frac{\sigma }{S},\;\;\text{LOQ}=10\frac{\sigma }{S}, \end{array}$$

where *σ* is the standard deviation of the intercept (115.82 ± 3300) of regression line and *S* is the slope (30875.64 ± 248.86) of regression line of the calibration curve [15]. The LOD and LOQ values were 0.081 and 0.242 *μ*g/mL, respectively.

#### 3.2.3. Accuracy and Precision

Accuracy and precision were determined by the recovery study of known amounts in accordance with ICH recommendation [15]. Five consecutive injections of STP sample solutions (2, 6, 10, 15, and 20 *μ*g/mL) showed excellent intraday accuracy (98.74–101.64%) and precision (0.64–2.32%) (Table 2). Interday accuracy and precision data of back-calculated concentration of calibration samples for STP were evaluated in triplicates by recovery studies using the standard addition method (Table 3).

#### 3.2.4. Robustness and Ruggedness

In order to measure the extent of the method robustness, the most critical parameters were interchanged while keeping the other parameters unchanged, and in parallel the chromatographic profile was observed and recorded. Robustness of the proposed method was determined using STP concentration of 10 *μ*g/mL and changing the flow rate (0.9–1.1 mL/min) and column temperature (±2°C) and change in the pH of mobile phase (±0.2). No significant changes in assay value were observed (Table 3) by changing the chromatographic conditions which confirms the robustness of the method. Ruggedness of the method was determined by using mobile phase components from two different manufactures and changing analysts. There was no significant change in the retention time of STP which was observed; %RSD was 0.31–0.63% indicating the ruggedness of the method.

#### 3.2.5. Sample Solution Stability

The stability of the drug in solution during analysis was determined by repeated analysis of samples during the course of experimentation on the same day and also after storage of the drug solutions (calibration samples) for 24, 48, and 72 hours under laboratory bench condition (25 ± 1°C) and under refrigeration (4.0 ± 0.5°C). There was no significant change in analysis over a period of 72 hours. The mean %RSD between peak areas for the samples stored under refrigeration (4.0 ± 0.5°C) and at laboratory temperature (25 ± 1°C) was found to be 0.58% and 0.93%, respectively, suggesting that the drug solution can be stored without any degradation over the time interval studied.

### 3.3. Stability-Indicating Study

**Figure 3:**
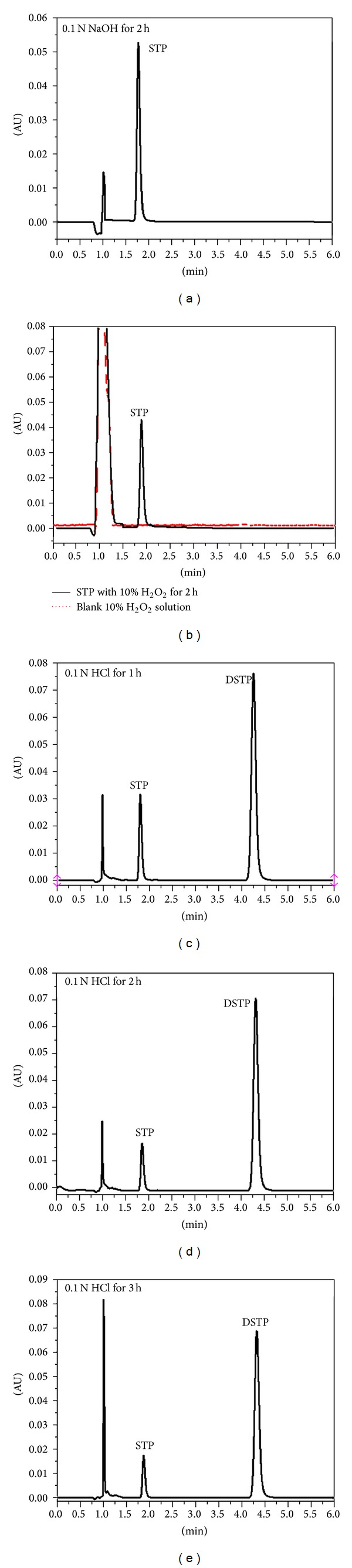
Representative chromatograms of stress testing of STP. (a) STP subjected to 0.1 N NaOH forced degradation for 2 h; (b) STP subjected to 10% H_2_O_2_ oxidation for 2 h; and (c)–(e) STP subjected to 0.1 N HCl degradation for 1, 2, and 3 h, respectively. Chromatograms of the samples that have been subjected to thermal and photolytic stress conditions are identical with (a).

The ICH guideline entitled stability testing of drug substances and products [15] requires the stress testing to be carried out to elucidate the inherent stability characteristics of the active substance and provide a rapid identification of differences that might result from changes in the manufacturing processes or source sample. Susceptibility to oxidation and hydrolytic and photolytic stability are the required tests. An ideal stability-indicating method is one that quantifies the standard drug alone and also resolves its degradation products. As described in the experimental section, different stress conditions were applied: boiling, acid/base forced degradation, oxidation, and irradiation with UV light. From this investigation, it was clear that, in case of boiling, base-forced degradation (1 N NaOH), oxidation (H_2_O_2_), and UV irradiation (solid state and solution), STP was stable under the employed stress conditions as no degradation products were observed in their chromatograms (Figures 3(a) and 3(b)) which confirms the data from European Medicines Agency (EMEA) [10]. However, in case of acidic conditions, one degradation product was observed, DSTP, at a retention time of 4.25 min (peak resolution from STP peak was 5.89) (Figures 3(c)–3(e)). The method was able to separate completely the degradation product from the intact STP. This confirmed the selectivity and stability-indicating property of the proposed method. The intact STP concentration was calculated and found to be degraded by 76.03, 83.51, 84.87, and 95% in case of acid degradation using 0.1 N hydrochloric acid after 1, 2, 3, and 5 hours, respectively.

### 3.4. Structure Elucidation of Acidic Degradation Product (DSTP)

**Scheme 1:**
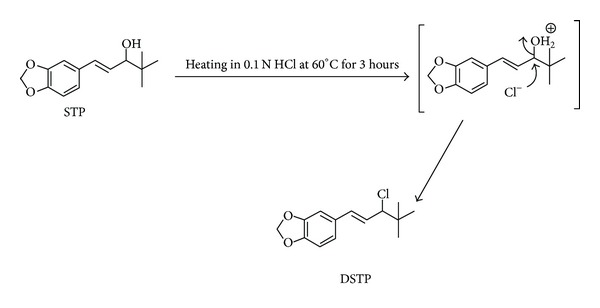
Postulated acidic degradation pathway of STP.

**Figure 4:**
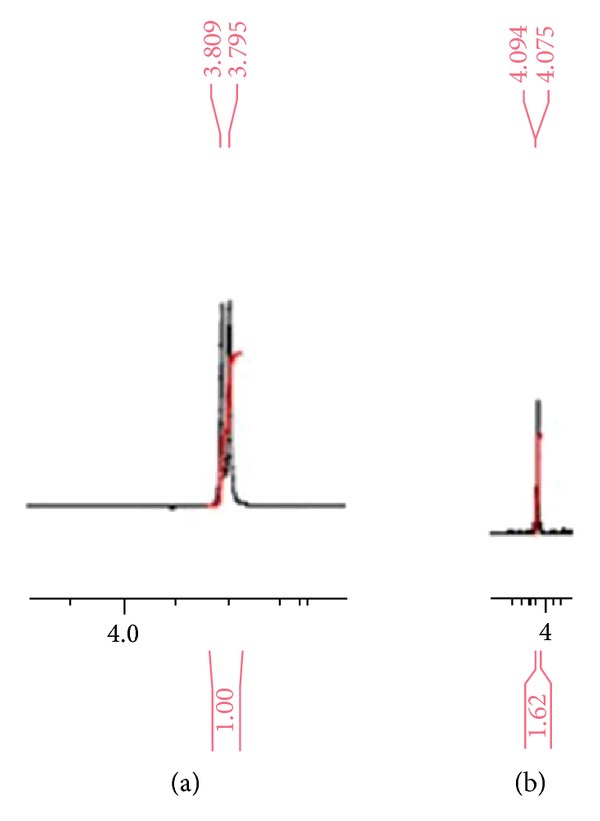
(a) Segment of ^1^H-NMR spectrum of STP; (b) segment of ^1^H-NMR spectrum of DSTP.

**Figure 5:**
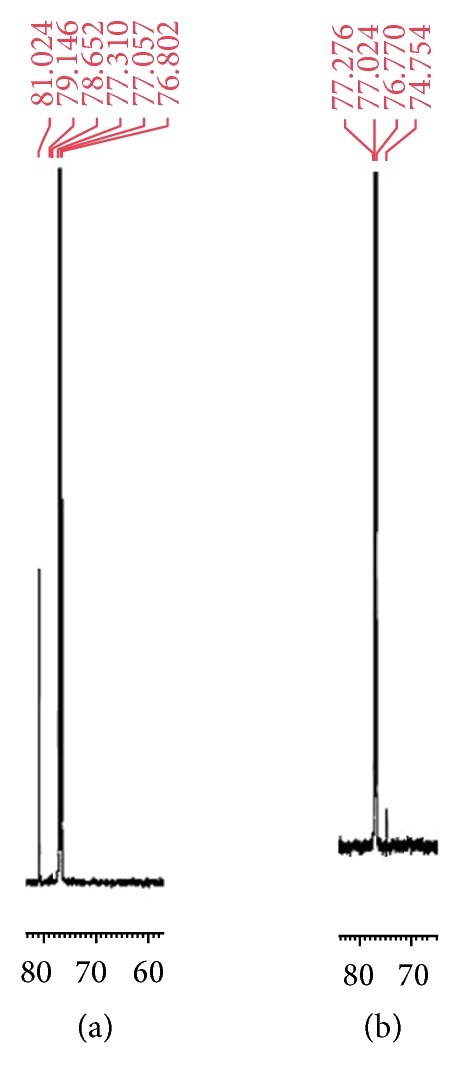
(a) Segment of ^13^C-NMR spectrum of STP; (b) segment of ^13^C-NMR spectrum of DSTP.

Acidic degradation of STP was studied using ^1^H-NMR, ^13^C-NMR (Figures 4 and 5), and mass spectral data. ^1^H-NMR spectrum of DSTP showed a signal integrating to one proton at 4.09 ppm which was assigned to be Cl–CH with concomitant disappearance of the signal of OH functionality at 1.56 ppm and the signal of the methine proton (OH–CH) at 3.80 ppm. Additionally, ^13^C-NMR of DSTP showed signal at 74.8 ppm which was assigned to be Cl–CH with concomitant disappearance of the signal of OH–CH at 83.9 ppm. Moreover, mass spectrum (ESI) of DSTP showed peak at* m/z* 217 [M-36 + H]^+^. UV spectra of STP (Figure 1) and DSTP are identical indicating that there is no change in the conjugation system upon this acidic degradation of STP. Structure elucidation of DSTP explained its delaying in HPLC elution due to its lower polarity compared to STP. Accordingly, the aforementioned analytical data of DSTP suggested that upon acid degradation of STP the hydroxyl moiety was protonated with hydrochloric acid and subsequently substituted with chloride ion to give the halogenated derivative DSTP (Scheme 1). ^1^H-NMR (CDCl_3_) of STP: *δ* (ppm) = 0.88 (s, 9H, *t*-butyl), 1.56 (s, 1H, OH), 3.80 (d, *J* = 7.0 Hz, 1H, CHOH), 5.86 (s, 2H, OCH_2_O), 6.05 (dd, *J* = 15.7, 7.85 Hz, 1H, CH=CH–C_6_H_3_), 6.39 (d, *J* = 15.5 Hz, 1H, CH=CH–C_6_H_3_), 6.67 (d, *J* = 8.0 Hz, 1H, Ar–H), 6.72 (d, *J* = 7.95 Hz, 1H, Ar–H), 6.85 (s, 1H, Ar–H). ^13^C-NMR (CDCl_3_) of STP: *δ* (ppm) = 26.5 (C(CH_3_)_3_), 32.8 (C(CH_3_)_3_), 81.9 (CH–OH), 101.1 (OCH_2_O), 107.1, 108.3, 121.1 (Ar–CH), 126.9, 131.5 (CH=CH–C_6_H_3_, CH=CH–C_6_H_3_, Ar–C), 147.2, 148.0 (Ar–C). MS* m/z* (ESI): 217 [M-18 + H]^+^.

^1^H-NMR (CDCl_3_) of DSTP: *δ* (ppm) = 0.83 (s, 9H,* t*-butyl), 4.09 (d, *J* = 9.5 Hz, 1H, CH–Cl), 5.74 (s, 2H, OCH_2_O), 5.92 (dd, *J* = 15.4, 7.95 Hz, 1H, CH=CH–C_6_H_3_), 6.23 (d, *J* = 15.5 Hz, 1H, CH=CH–C_6_H_3_), 6.54 (d, *J* = 8.0 Hz, 1H, Ar–H), 6.60 (d, *J* = 7.96 Hz, 1H, Ar–H), 7.04 (s, 1H, Ar–H). ^13^C-NMR (CDCl_3_) of DSTP: *δ* (ppm) = 26.8 (C(CH_3_)_3_), 36.4 (C(CH_3_)_3_), 74.8 (CH–Cl), 101.2 (OCH_2_O), 105.9, 108.3, 121.6 (Ar–CH), 125.7, 130.6, 132.5 (CH=CH–C_6_H_3_, CH=CH–C_6_H_3_, Ar–C), 147.8, 148.1 (Ar–C). MS* m/z* (ESI): 217 [M-36 + H]^+^.

### 3.5. Kinetics of Degradation in Acidic Condition

**Figure 6:**
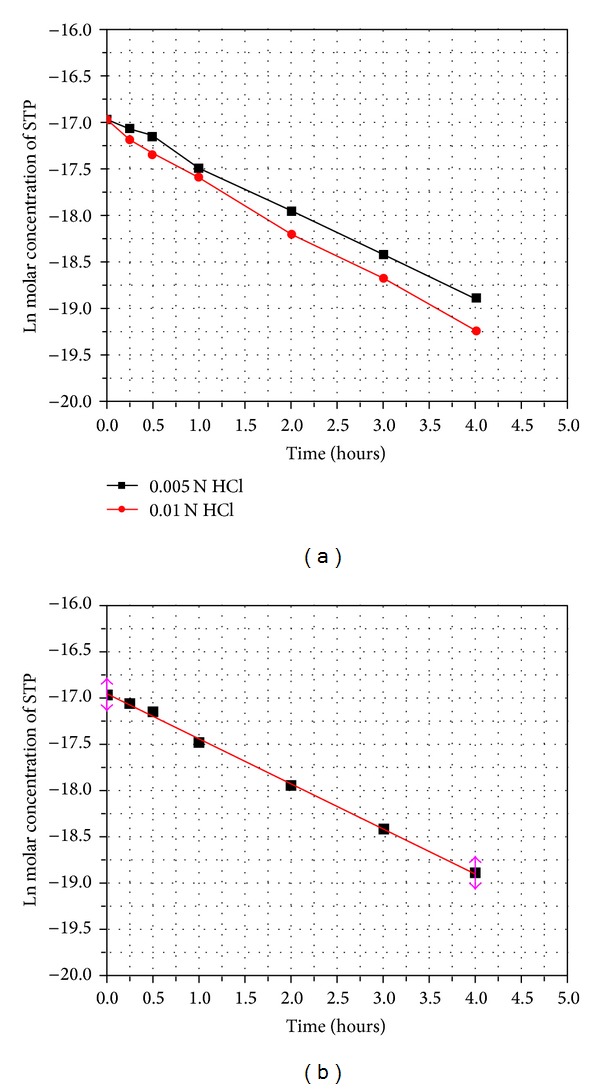
(a) Plot of the effect of HCl concentration on the rate of degradation of STP; (b) kinetic plot of the acidic degradation of STP using 0.01 N HCl.

The degradation of STP was investigated in acidic condition using 0.005, 0.01, 0.05, 0.1 N HCl. The drug was found to be extremely labile for higher concentrations of HCl (0.05 and 0.1 N). A gradual decrease in STP concentration was observed with increasing time at lower HCl concentrations (0.005 and 0.01 N) (Figure 6(a)). A linear relationship was observed by plotting the 1og of residual molar concentration of STP versus time (Figure 6(b)) which indicates first-order kinetics according to the following equation:

(2) $$\begin{array}{c} \ln C_{t}=-kt+\ln C_{0}. \end{array}$$

Here, *C*_*t*_ is the remaining STP molar concentration, *C*_0_ is the initial molar concentration of STP, *k* is the apparent first order rate constant with a negative sign, and *t* is the time. The plot gives a slope of −*k* of 0.4882 mol/h. Good correlation coefficient was observed (*r* ^2^ = 0.9982). The half-life (*t*_1/2_) of the first-order reaction was found to be *t*_1/2_ = 1.42 h according to the following equation:

(3) $$\begin{array}{c} t_{1/2}=\frac{\ln\left(2\right)}{k}. \end{array}$$

### 3.6. Applicability of the Method

It is evident from the results obtained previously that the proposed method gave satisfactory results with the analysis of STP in bulk. Thus, STP-containing capsules were subjected to the analysis by the proposed method. The same procedure for determination of drug dosage form was performed. The peak area of five injections was compared by a standard solution of STP with the same concentration level and the mean recovery percentage was calculated to be 99.34 ± 0.59%. This acceptable value indicated the applicability of the method for the routine quality control of STP capsules without interference from the excipients. This was evidenced from the good label claim percentage as well as the absence of any peaks in the chromatogram of the capsule extract solution (Figure 2).

## 4. Conclusions

The present study represents the first report that deals with the development and validation of a stability-indicating HPLC-DAD method for determination of STP. This study is a typical example of development of a stability-indicating assay, established following the recommendations of ICH guidelines. The proposed method showed acceptable accuracy, precision, and selectivity. From the economical point of view, the method involved the native UV-absorbing property of STP, rather than expensive derivatizing analytical reagents. STP showed great stability at the various stress conditions (thermal, alkaline, oxidative, and photodegradation). A single degradation product, DSTP, was only formed with the exposure of STP to acidic degradation using HCl. Structure elucidation of the acidic degradation product using ^1^H-NMR, ^13^C-NMR, and ion trap mass spectrometry revealed substitution of OH functionality of STP with chloride following treatment of STP with hydrochloric acid. Additionally, kinetic study of the acidic degradation reaction demonstrated that STP undergoes first-order kinetics. The method is suitable for the determination of STP in bulk and capsule form without any interference from the degradation products, and it is recommended for routine use in quality control industry laboratories.

## Figures and Tables

**Table 1:** Summary of forced degradation conditions for STP.

Stress conditions			Exposed conditions	Duration
Thermal stress	Solid/Solution	—	80°C	48 h

Acid/Base degradation	Acid	0.005, 0.01, 0.05, and 0.1 N HCl	60°C	1, 2, and 3 h
	Base	0.05, 0.1, 0.5, and 1 N NaOH	60°C	1, 2, and 3 h

Oxidation	H_2_O_2_	1, 6, 10, and 30%	Room temperature	2 h

Photodegradation	Solid/Solution	UV (254 nm)	Room temperature	72 h

**Table 2:** Intraday (*n* = 3) and interday (*n* = 3) precision and accuracy of STP.

Nominal concentration (*μ*g/mL)	Intraday				Interday			
	Mean ± SD (*μ*g/mL)	Accuracy (%)	RSD (%)	Bias (%)	Mean ± SD (*μ*g/mL)	Accuracy (%)	RSD (%)	Bias (%)
2	2.01 ± 0.04	100.68	2.03	0.5	1.95 ± 0.09	97.78	1.41	−2.5
6	6.10 ± 0.04	101.64	0.64	1.67	6.02 ± 0.07	99.90	1.18	0.33
10	9.87 ± 0.18	98.74	1.76	−1.3	9.99 ± 0.40	99.75	2.65	−0.1
15	14.85 ± 0.35	98.99	2.32	−1.0	14.95 ± 0.42	99.85	2.32	−0.3
20	20.07 ± 0.39	100.36	1.94	0.35	19.95 ± 0.54	99.55	3.03	−0.2

**Table 3:** Robustness of the method.

Parameters changed	Variation	Retention time of STP (min)	Peak area∗	Bias (%)
Temperature (°C)	23	1.81	303409	−0.31
	27	1.80	312560	2.69

Flow rate (mL/min)	0.9	1.83	308654	1.41
	1.1	1.77	314565	3.35

pH	3.9	1.79	312468	2.66
	4.0	1.78	296265	−2.66
	4.2	1.84	304648	0.092
	4.3	1.82	289216	−4.98

*Peak area of 10 *μ*g/mL of STP.
